# Identifying Patients With Delirium Based on Unstructured Clinical Notes: Observational Study

**DOI:** 10.2196/33834

**Published:** 2022-06-24

**Authors:** Wendong Ge, Haitham Alabsi, Aayushee Jain, Elissa Ye, Haoqi Sun, Marta Fernandes, Colin Magdamo, Ryan A Tesh, Sarah I Collens, Amy Newhouse, Lidia MVR Moura, Sahar Zafar, John Hsu, Oluwaseun Akeju, Gregory K Robbins, Shibani S Mukerji, Sudeshna Das, M Brandon Westover

**Affiliations:** 1 Massachusetts General Hospital Boston, MA United States

**Keywords:** delirium, electronic health records, clinical notes, machine learning, natural language processing

## Abstract

**Background:**

Delirium in hospitalized patients is a syndrome of acute brain dysfunction. Diagnostic (International Classification of Diseases [ICD]) codes are often used in studies using electronic health records (EHRs), but they are inaccurate.

**Objective:**

We sought to develop a more accurate method using natural language processing (NLP) to detect delirium episodes on the basis of unstructured clinical notes.

**Methods:**

We collected 1.5 million notes from >10,000 patients from among 9 hospitals. Seven experts iteratively labeled 200,471 sentences. Using these, we trained three NLP classifiers: Support Vector Machine, Recurrent Neural Networks, and Transformer. Testing was performed using an external data set. We also evaluated associations with delirium billing (ICD) codes, medications, orders for restraints and sitters, direct assessments (Confusion Assessment Method [CAM] scores), and in-hospital mortality. F1 scores, confusion matrices, and areas under the receiver operating characteristic curve (AUCs) were used to compare NLP models. We used the φ coefficient to measure associations with other delirium indicators.

**Results:**

The transformer NLP performed best on the following parameters: micro F1=0.978, macro F1=0.918, positive AUC=0.984, and negative AUC=0.992. NLP detections exhibited higher correlations (φ) than ICD codes with deliriogenic medications (0.194 vs 0.073 for ICD codes), restraints and sitter orders (0.358 vs 0.177), mortality (0.216 vs 0.000), and CAM scores (0.256 vs –0.028).

**Conclusions:**

Clinical notes are an attractive alternative to ICD codes for EHR delirium studies but require automated methods. Our NLP model detects delirium with high accuracy, similar to manual chart review. Our NLP approach can provide more accurate determination of delirium for large-scale EHR-based studies regarding delirium, quality improvement, and clinical trails.

## Introduction

Delirium is an acute neuropsychiatric syndrome with features of inattention and global cognitive dysfunction, associated with increased hospital length of stay, in-hospital mortality, and long-term cognitive disability [1]. Delirium occurs in up to 26% of hospitalized patients; prevalence rates may reach 42% in patients older than age 65 years [2].

Electronic health records (EHRs) offer a rich source of information for studies of delirium; however, determining which patients have delirium is challenging. Manual review of medical records is time consuming, limiting studies to a small fraction of patients at risk. A more scalable approach is to use International Classification of Diseases (ICD) billing codes. This approach was recently used by a study [3] to assess 200 patients admitted to a skilled nursing facility, revealing that ICD codes achieved 96.0% specificity but only 53.1% sensitivity. Another study [4] analyzed clinical data from 184 older adults at one academic medical center and found that ICD codes had a specificity of 98% and sensitivity of 18%. Thus, ICD codes miss a large fraction of patients with delirium.

On the other hand, rich information about patients’ status exists in narrative clinical notes from doctors, nurses, physical therapists, and other health care workers [5]. However, extracting this information is challenging because of the flexibility of natural language.

In this work, we collected 1.5 million clinical notes from over 10,000 patients from 7 distinct cohorts from among 9 hospitals and developed a natural language processing (NLP) algorithm to identify patients with delirium from unstructured EHR notes.

## Methods

### Data Set Description and Sentence Extraction

We collected 1,565,678 clinical notes from 10,516 patients from 9 hospitals, including Massachusetts General Hospital, Brigham and Women's Hospital, Cooley Dickinson Hospital, Martha's Vineyard Hospital, McLean Hospital, Nantucket Cottage Hospital, Newton-Wellesley Hospital, North Shore Medical Center, and Spaulding Rehabilitation Hospital. These 10,516 patients were from 7 previously assembled cohort studies:

- Antiepileptic drug (AED) data set: this data set comprises patients who received AEDs and is used to study adverse effects of AEDs (n=852).
- GIFTS data set: this data set comprises older patients admitted for orthopedic surgery and is used to study delirium (n=576).
- Dementia data set: this data set comprises patients who were at risk for dementia and is used to study dementia (n=802).
- COVID-19 data set: this data set comprises patients who were hospitalized for COVID-19 and is used to study hospitalization, intensive care unit admission, intubation, and mortality prediction for patients with COVID-19 (n=3429).
- NCC data set: this data set is used to study neurological diseases such as delirium, headache, and anosmia for patients at neurocritical care units (n=1985).
- LTM data set: this data set comprises acutely ill patients undergoing continuous electroencephalographic monitoring (n=395). These patients underwent in-person delirium assessments by research staff. Thus, this data set contains assessment records rather than clinical notes.
- Control data set: this data set comprises inpatients randomly selected as a control group from the Massachusetts General Brigham hospital system (n=2477).

Demographic features of these cohorts are shown in Multimedia Appendix 1.

### Creating the Gold Standard: Sentence Labeling

We first created a comprehensive collection of keywords related to delirium; these included the following: “delirium,” “delirious,” “encephalopathy,” “confused,” “confusion,” “agitated,” “agitation,” “inattentive,” “inattention,” “disorient,” “disoriented,” “disorientation,” “reorient,” “restraints,” “lethargy,” “psychosis,” “hallucination,” “inappropriate behavior,” “fluctuating arousal,” “altered mental status,” “mental status change,” “fluctuating mental status,” and “waxing and waning mental status.” We extracted all sentences containing any of these keywords from the assembled collection of notes.

Next, we created a gold-standard set of labels for sentences. Examples are shown in Multimedia Appendix 2.

We developed a graphical user interface (GUI) for efficient iterative labeling of sentences. Active learning, an algorithm to select the most informative samples, was used to select candidate sentences in each round. The labeling process was as follows:

- Step 0: candidate sentences were randomly selected from the set of unlabeled sentences.
- Step 1: experts labeled candidate sentences and created regular expressions called “always patterns” (described below in Regular Expression Generation).
- Step 2: unlabeled sentences were screened for “always patterns,” corresponding labels were assigned to sentences that match, and these were added to the labeled set.
- Step 3: the labeled sentences were used to train a classifier (introduced in Prediction Model).
- Step 4: the classifier was used to scan unlabeled sentences and assign them a label and an embedding vector.
- Step 5: sentence embedding vectors were used to generate an embedding map via Uniform Manifold Approximation and Projection [6].
- Step 6: candidate sentences were selected from the unlabeled data set with two query strategies: uncertainty based on the entropy of prediction scores and diversity based on the embedding map (Multimedia Appendix 3). Each query selects half of the candidate sentences for the next round. Then, the process was reverted to step 1.

### Regular Expression Generation

While labeling sentences, experts created “always patterns”: a regular expression that, when present, warrants assigning the corresponding label to the sentence. Multimedia Appendix 2 provides examples of “always patterns” for positive, negative, and neither patterns. The GUI used “always patterns” to scan the residual unlabeled sentences to assign a label to all matched sentences, thus enhancing labeling efficiency.

### Prediction Model

We developed three models to identify delirium sentences: Support Vector Machine (SVM), long short-term memory (LSTM), and Transformer models. The LSTM model was also used in active learning when collecting labels. Details of the three models are as follows.

SVM is a widely used text classifier based on a “bag of words” representation [7]. Sentences with delirium-related keywords are first transformed into sentence vectors via “a bag of unigrams and bigrams,” and the SVM algorithm finds hyperplanes that separate different categories. The distances between sample points and hyperplanes are used to calculate prediction scores.

Recurrent neural networks with LSTM units (RNN-LSTM) are common models for sequence learning, where an LSTM unit contains a cell for memory, an input gate to control input information flow, an output gate to control output information flow, and a forgetting gate to update memory [8]. We used a 3-layer bidirectional RNN with LSTM units to encode sentences. The vector representation corresponding to the keyword location was used for classification.

A transformer is a previously proposed [9] transduction model that computes a representation of each word in a sentence relying on self-attention. It is also the model used in Bidirectional Encoder Representations from Transformers (BERT) [10]. We used a 3-layer Transformer model to transform a sentence into a sequence of vectors. The vector representation corresponding to the delirium keyword was then used for classification. The word vectors from BERT were used as initial vectors.

### Comparison of Delirium NLP Results With Other Delirium Indicators

To evaluate construct validity of our EHR-based delirium detection algorithms, we evaluated the strength of the association between presence of delirium as detected by our NLP models with other clinical outcomes or events known to be associated with delirium. These included the use of ICD billing codes for delirium; use of medications related to delirium; use of restraints and sitters; and in-hospital mortality. For one cohort (the LTM data set) we had access to one-time in-person delirium assessments using the Confusion Assessment Method (CAM), which has been already been validated as a good proxy for DSM-5 in prior studies. For these, we compared the presence of delirium, as defined by CAM, with the presence of positive delirium sentences in clinical notes during hospitalization. Details are provided in Multimedia Appendix 4.

### Interrater Agreement

Pairwise interrater agreement (IRA) is used to measure agreement between human and human (model) for each category. Details are provided in Multimedia Appendix 5.

### Data Split for Evaluation

We combined the AED, GIFTS, Dementia, COVID-19, NCC, and Control data sets to yield a data set for sentence labeling based on active learning. We collected 200,471 labeled sentences, including those directly labeled by human experts and those matched by “always patterns.” Of the 200,471 labeled sentences, 176,800 were “positive,” 15,577 were “negative,” and 8094 were “neither” sentences.

We designed two types of tests for NLP delirium detection algorithms: an internal test and an external test (see Multimedia Appendix 6).

#### Internal Test

In the internal test, we followed the standard machine learning evaluation pipeline, randomly splitting the 200,471 labeled sentences into a training data set (120,283 sentences, 60%), validation data set (40,094 sentences, 20%) for hyperparameter tuning, and test data set (40,094 sentences, 20%) for performance evaluation.

#### External Test

The LTM data set was not used for training the NLP algorithms. It was used entirely for testing. The LTM data set contained 16,067 sentences: 14,378 positive, 1193 negative, and 496 neither sentences.

### Data Security and Ethics Approval

We have ethics approval (2013P001024) from the MassGeneral Brigham institutional review board to work with identified data internally. We will deidentify the data for sharing them with external partners to test and improve the models together. Some existing deidentification algorithms have been developed, such as the Phsyionet algorithm [11] and the Philter algorithm [12], but the recall of these algorithms is close to 100% rather than 100% perfect. Another option is federated learning, namely training the model across multiple decentralized machines holding local data by us and our external partners, without exchanging them.

## Results

### Performances of Delirium NLP classifiers

In the following analysis, the 95% CIs were calculated through bootstrapping [13].

Table 1 compares performances of SVM, RNN-LSTM, and Transformer on both internal and external tests. As the data set is an imbalanced multiclass data set, micro F1 scores, and macro F1 scores were used to evaluate performance [14]. When using micro F1 scores, the performance of the SVM, RNN-LSTM, and Transformer models was close on both the internal and external test sets. However, when using macro F1 scores, which measure average performance across categories, on the internal test the Transformer (0.927, 95% CI 0.925-0.930) performed similarly to the RNN-LSTM (0.922, 95% CI 0.920-0.925), and both Transformer and RNN-LSTM outperformed the SVM (0.839, 95% CI 0.835-0.842). In the external test set, the Transformer (0.918, 95% CI 0.914-0.921) displayed the best performance, while the SVM (0.885, 95% CI 0.881-0.889) displayed slightly better performance than the RNN-LSTM (0.868, 95% CI 0.862-0.874). Overall, the Transformer was thus the best model based on both micro F1 and macro F1 metrics.

**Figure 1:**
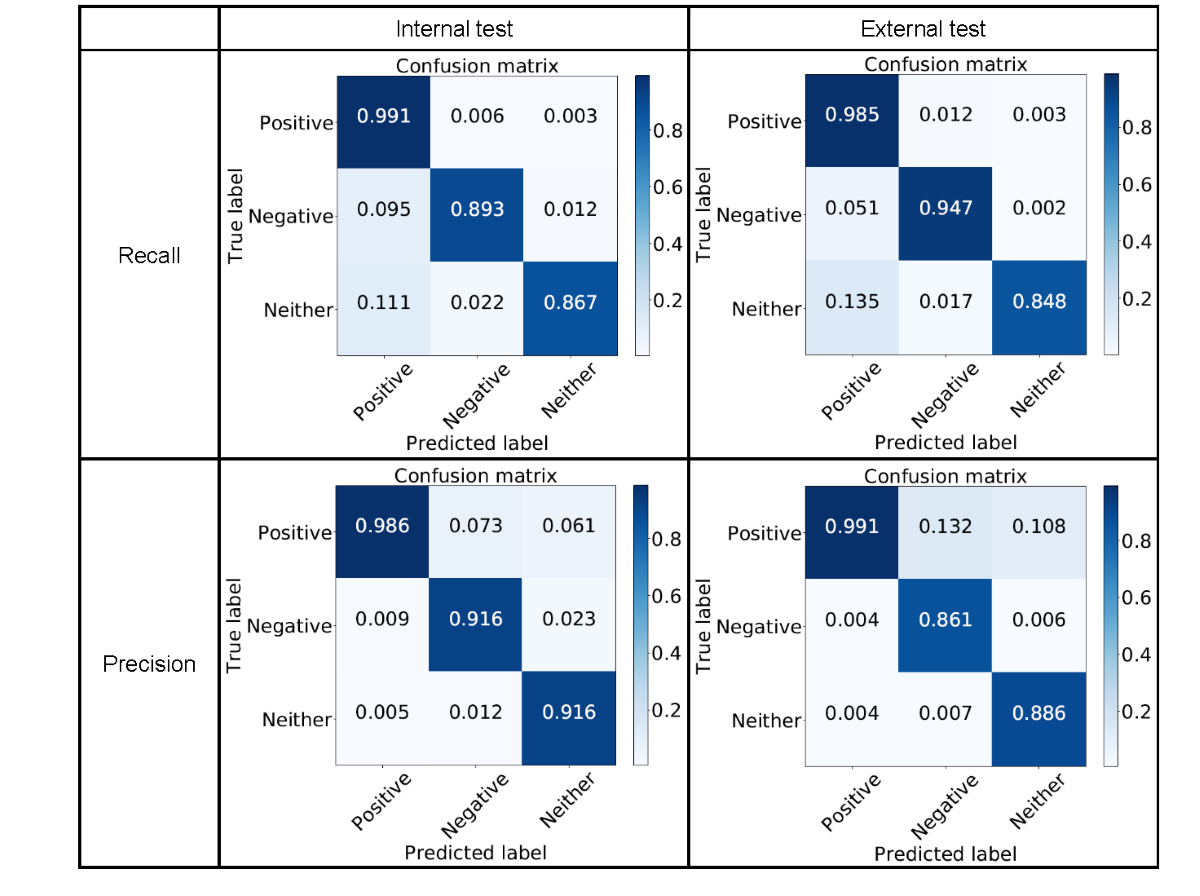
Precision, recall, and F1 scores for delirium classifiers.

Figure 1 illustrates confusion matrices for the best Transformer, normalized by row to show recall (sensitivity), and by column to show precision (positive predictive value). For the Positive category, precision and recall on both the internal and external test were close to 0.99. For the Negative category, on the internal test, precision (0.916, 95% CI 0.911-0.920) was slightly higher than recall (0.893, 95% CI 0.889-0.897), while on the external test, recall (0.947, 95% CI 0.942-0.951) was much higher than precision (0.861, 95% CI 0.852-0.870). For the Neither category, on both internal and external tests, precision (0.916, 95% CI 0.909-0.923 vs 0.886, 95% CI 0.877-0.894) was better than recall (0.867, 95% CI 0.860-0.873 vs 0.848, 95% CI 0.836-0.859). In summary, performance on the Negative category was better than that on the Neither category, and performance on the Positive category was better still.

**Figure 2:**
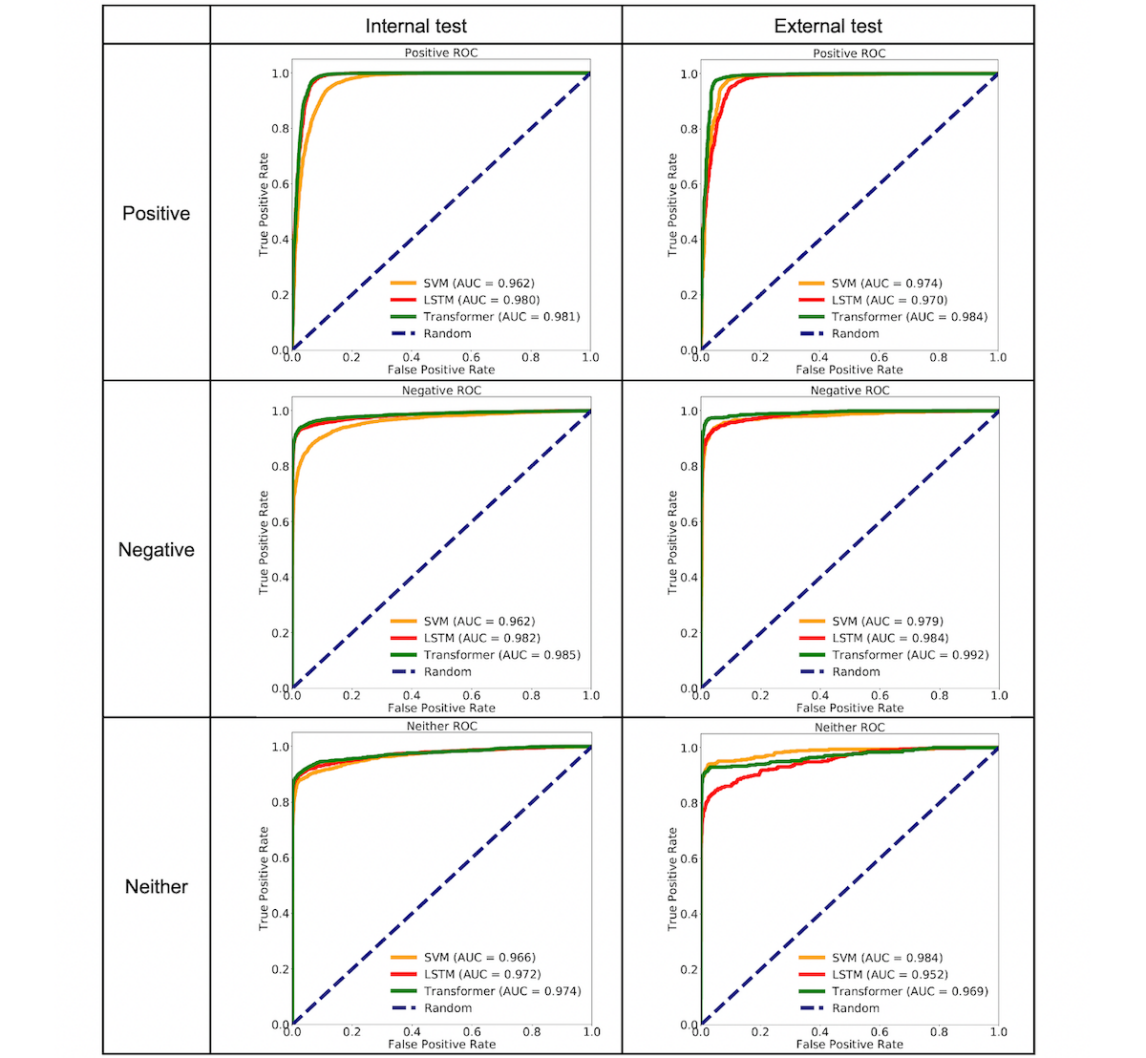
Receiver operating characteristic (ROC) curves for delirium classifiers. AUC: area under the curve, LSTM: long short-term model, SVM: Support Vector Machine.

Figure 2 compares receiver operating characteristic (ROC) curves and areas under the ROC curve (AUCs) for the Positive, Negative, and Neither categories on both internal and external tests. On the internal test data, the Transformer (Positive: 0.981, 95% CI 0.980-0.983; Negative: 0.985, 95% CI 0.984-0.986; Neither: 0.974, 95% CI 0.971-0.976) and RNN-LSTM (Positive: 0.980, 95% CI 0.978-0.981; Negative: 0.982, 95% CI 0.981-0.983; Neither: 0.972, 95% CI 0.969-0.974) were close, and both were better than SVM (Positive: 0.962, 95% CI 0.961-0.964; Negative: 0.962, 95% CI 0.961-0.963; Neither: 0.966, 95% CI 0.963-0.968).

On the external test, for the Positive category, the Transformer (0.984, 95% CI 0.983-0.985) was the best, and the SVM (0.974, 95% CI 0.972-0.976) was better than the RNN-LSTM (0.970, 95% CI 0.966-0.972). For the Negative category, the Transformer (0.992, 95% CI 0.991-0.993) was the best, followed by RNN-LSTM (0.984, 95% CI 0.982-0.985), and then the SVM (0.979, 95% CI 0.977-0.981). For the Neither category, the SVM (0.984, 95% CI 0.982-0.986) was the best, followed by the Transformer (0.969, 95% CI 0.967-0.973) and the RNN-LSTM (0.952, 95% CI 0.949-0.955).

We conclude that overall, the Transformer model performed the best. Hereinafter, “NLP” refers to the Transformer model.

**Table 1:** F1 scores for the Support Vector Machine, recurrent neural networks with long short-term model, and the Transformer model.

Scores		Support Vector Machine, mean (95% CI)	Recurrent neural networks with long short-term model, mean (95% CI)	Transformer, mean (95% CI)
**Micro F1**				
	Internal test	0.949 (0.948-0.951)	0.977 (0.976-0.978)	0.978 (0.977-0.979)
	External test	0.964 (0.963-0.966)	0.967 (0.965-0.968)	0.978 (0.977-0.979)
**Macro F1**				
	Internal test	0.839 (0.835-0.842)	0.922 (0.920-0.925)	0.927 (0.925-0.930)
	External test	0.885 (0.881-0.889)	0.868 (0.862-0.874)	0.918 (0.914-0.921)

### Associations Between Delirium NLP Results and Other Delirium Indicators

Next, we compared associations between delirium NLP results and other delirium indicators. Results are shown in Table 2 For the NCC cohort (n=1985 patients), we assessed associations of NLP-detected delirium with delirium ICD code usage, medications, restraints and sitter orders, and mortality. For the LTM data set (n=395), we analyzed associations with CAM scores. For comparison, we also calculated the association of ICD code usage with the same delirium indicators.

We calculated these delirium indicators at the patient level, such that each patient is assigned a “+1” for NLP-based detection of delirium if they have one or more sentences classified as Positive by the NLP Transformer algorithm; otherwise, they were assigned a “–1.” Similarly, patients were assigned scores of “+1” or “–1” for each of the other delirium indicators. We used the φ coefficient (mean square contingency coefficient) to measure associations between NLP-based delirium detections and each delirium indicator. When using our NLP detector to classify sentences in the NCC (or LTM) data set, the NCC (or LTM) data were only used as test data, as illustrated in Multimedia Appendix 6.

Table 2 shows that associations of delirium indicators with NLP results are much stronger than those with ICD codes.

In the NCC data set, the NLP model identified 1117 out of 1985 patients with positive delirium sentences (which were verified to be correct through manual review) but no delirium ICD codes. This highlights the low sensitivity of delirium ICD codes relative to manual chart review, and the excellent sensitivity of the NLP algorithm.

**Table 2:** Associations between delirium natural language processing indicators and other delirium indicators.

Data sets and delirium indicators		International Classification of Diseases codes, mean (95% CI)	Natural language processing classifiers, mean (95% CI)
**NCC**			
	International Classification of Diseases codes	1	0.134 (0.133 to 0.135)
	Medication	0.073 (0.072 to 0.074)	0.194 (0.192 to 0.197)
	Restraints and sitter orders	0.177 (0.176 to 0.179)	0.358 (0.357 to 0.361)
	Mortality	0.000 (–0.0002 to 0.0001)	0.216 (0.215 to 0.217)
**LTM**			
	Confusion Assessment Method	–0.028 (–0.025 to –0.030)	0.256 (0.252 to 0.259)

### Coverage Analysis

In creating the gold standard for labeling sentences, we developed many “always patterns” for delirium. While this set of sentences was large, we hypothesized that it might not be exhaustive; therefore, we investigated the coverage of our “always patterns” in another data set.

We analyzed the coverage of “always patterns” as follows. First, in the development data set (AED, GIFTS, Dementia, COVID-19, NCC, and control cohorts)—used for labeling the gold-standard set of sentences and for developing “always patterns”—97.6% (195,680) of sentences with delirium keywords were matched by at least one “always pattern.” In the LTM data set, which was not used for labeling sentences, 78.2% (12,569) of sentences with delirium keywords matched at least one “always pattern.”

We next tested the extent to which sentences not matched by “always patterns” were still accurately classified by the NLP model. To accomplish this, we randomly selected 400 sentences as follows:

- 100 sentences that both the Transformer and LSTM models predicted “Positive” for delirium
- 100 sentences that both the Transformer and LSTM models predicted “Negative” for delirium
- 100 sentences that both the Transformer and LSTM models predicted “Neither”; namely, not relevant to delirium
- 100 sentences on which the Transformer and LSTM models disagreed.

**Figure 3:**
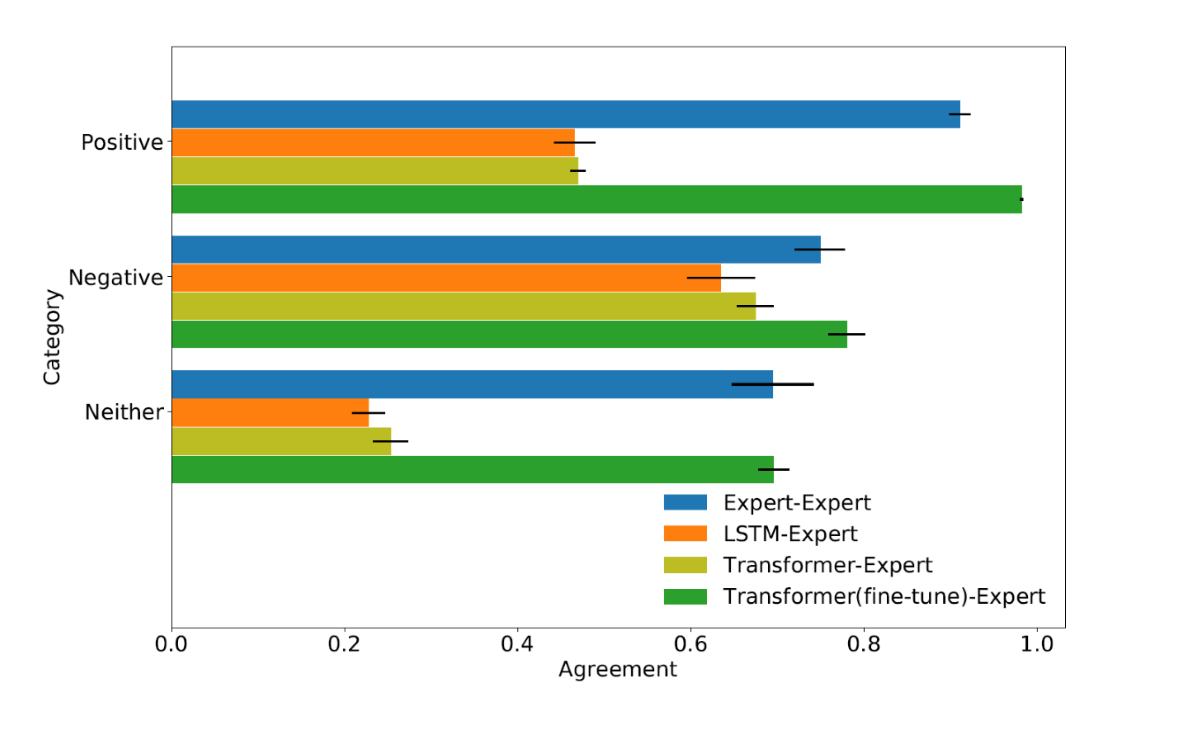
Pairwise interrater agreement (IRA) for unmatched sentences. LSTM: long short-term memory.

Two human experts (SM and MBW) independently labeled these 400 unmatched sentences. Pairwise IRA results are shown in Figure 3, where 95% CIs were calculated through Bootstrapping [13]. For unmatched sentences, the performance of model IRA (LSTM, Transformer) was close to that of human IRA for the Negative category but displayed gaps for Positive and Neither categories compared with human IRA.

We next investigated whether performance gaps in the new data set could be easily removed without repeating a large amount of sentence relabeling. For this investigation, we tried fine-tuning the Transformer model with a previously reported procedure [10]. This was readily done (green bars).

We conclude that the Transformer model is quite general, but not exhaustive; nevertheless, when gaps are encountered, the model can be readily tuned to accommodate previously unseen delirium sentence patterns.

**Figure 4:**
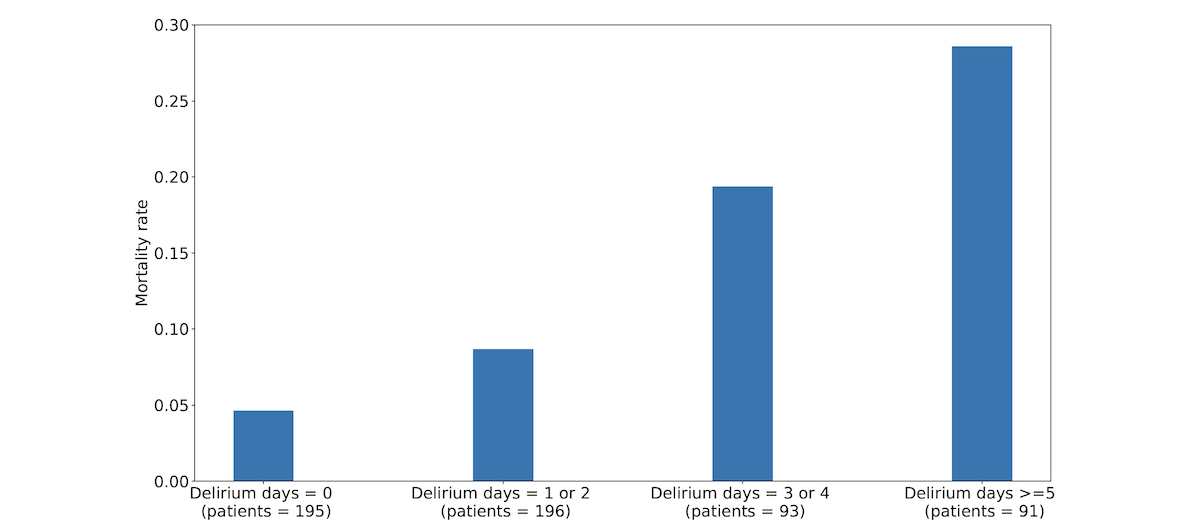
Mortality rate versus the number of days with delirium.

Figure 4 illustrates mortality rates for the patients with different numbers of days with delirium in the GIFTS data set. The mortality rate increases monotonically with the number of delirium days.

## Discussion

### Principal Findings

Our results show that an NLP approach can accurately detect patients with delirium, using unstructured clinical notes. These results are likely to be robust because they are based on a large collection of clinical notes from over 10,000 patients. The proposed delirium NLP approach is much more accurate, and especially more sensitive, than delirium ICD codes; it was able to detect patients who have delirium described in clinical notes but have no delirium ICD codes in their medical records. Further enhancing validity, NLP delirium detections are strongly associated with clinical factors known to be associated with delirium, including delirium-associated medications, use of restraints, and in-hospital mortality. This NLP tool will be useful for large-scale EHR research on delirium.

### Application

The delirium NLP approach proposed in this work has many potential applications. First, the approach will be applied to many future large-scale studies regarding delirium, such as the causes of delirium and the effects of delirium on outcomes such as dementia. Second, the approach can review entire medical record in order to identify specific parts of the hospital, which seem to have more delirium, which can be used for quality improvement. We can use this to identify factors (eg, medications) that might explain why delirium occurs. Third, the approach can be used to develop a delirium prediction model for clinical trials. The detection results of the NLP approach can be used as targets of prediction models, and the prediction models can be used to identify patients at a high risk for delirium, which provides information for interventions. The barriers of the applications are data and trust or transparency.

### Comparison With Prior Work

Many prior studies have utilized ICD codes to identify delirium for large-scale EHR studies [3,4]. Our findings confirm observations from these earlier studies that ICD codes generally have high specificity but low sensitivity, leading to many missed cases of delirium. We investigated this finding in detail in the NCC cohort, where we observed that 1117 of 1985 patients who had positive delirium sentences had no corresponding delirium ICD codes. To confirm these findings, we used the NLP Transformer model to select the sentence with the highest positive score for each patient, and then manually reviewed the 1117 selected sentences, thereby manually confirming that these were true positives. These results show that the NLP approach largely overcomes the low sensitivity of delirium ICD codes.

NLP has been used to extract phenotypes from clinical notes in several previous studies. McCoy et al [15] used NLP to analyze discharge notes to improve prediction of suicide and accidental death after discharge. Gundlapalli et al [16] reported that a relatively simple case finding method based on string matching for specific keywords coupled with a negation algorithm and information extracted by a more complex NLP system could identify patients with inflammatory bowel disease. Zhou et al [17] applied an NLP approach to identify patients with depression on the basis of discharge summaries. Yang et al [18] explored transformer-based models for clinical concept extraction. Mascio et al [19] analyzed the impact of various word representations, text preprocessing, and classification algorithms on the performance of different text classification tasks based on EHRs. Most prior medical NLP used negation detection algorithms to deal with the negative cases. However, we found many negative cases that did not contain clear negative expressions. Therefore, we classified phenotype expressions as positive, negative, or neither (not relevant), and trained 3-class classifiers.

A few prior studies used NLP for delirium research. One such study [20] summarized patterns in the delirium literature over time, using unsupervised learning methods; by contrast, our work used NLP to extract information from clinical notes. Another study [21] detected delirium using an open-source NLP pipeline MedTaggerIE—an unstructured information management architecture–based information extraction framework. Shao et al [22] experimented with 3 different topic modeling methods and a keyword search method for identifying delirium-related documents and sentences in clinical notes. Weir et al [23] designed classifiers for patients with delirium by combining text data with ICD, Ninth Revision codes. Sun et al [24] defined a generic process for developing a clinical risk prediction model, applied the model calibration process at 4 hospitals, and generated risk prediction models for delirium. Jauk et al [25] implemented a random forest–based algorithm to identify hospitalized patients at high risk for delirium. A key difference between these prior studies and this study is that they aimed to detect delirium at the patient level (ie, whether a patient ever experienced delirium during a hospitalization). By contrast, our approach detects delirium at the sentence level, which provides more fine-grained temporal information (ie, on which days was a patient experiencing delirium). Such information is important for estimating the overall burden of delirium, and for studies that attempt to relate time-varying factors to the development of delirium.

### Strengths

This work leveraged a large cohort composed of multiple different cohorts. These data sets provide a good source for variety of delirium expression in clinical notes. Additionally, we developed a novel GUI labeling tool and used active learning to enhance labeling efficiency. Furthermore, we compared 3 widely used NLP classifiers including a state-of-the-art Transformer model for delirium detection. Finally, we compared our delirium NLP detector with other delirium indicators, and we were able to demonstrate that our NLP method is substantially better than traditional methods based on ICD codes.

### Limitations

Although our data were obtained from 9 hospitals, all were in the same geographic region (Massachusetts). Thus, our cohort may not be representative of other US or non-US populations. One important future direction is to test our delirium NLP algorithm using data from other regions. Additionally, the coverage rate of the “always pattern” for the development data set was 97.6% (n=195,680) owing to active learning, but decreased to 78.2% (n=12,569) on an independent test set. Further rounds of active learning to enlarge the available training data will help further expand the generalizability of the NLP Transformer model to new data sets. Nevertheless, our fine-tuning experiments show that extending the model to new data sets may require only a relatively small amount of additional labeling effort.

### Conclusions

In this work, we developed a new delirium NLP detection approach that identifies patients with delirium from unstructured clinical notes. In many cases, the delirium information was only recorded in clinical notes and was absent from ICD codes. We anticipate that this model will be useful for large-scale EHR-based research on delirium, especially detecting delirium at a fine-grained level such as the note and sentence levels. Additionally, the labeling process based on active learning developed for this study was very efficient, achieving a coverage rate of 97.6% (n=195,680) in the development data set after just 5 rounds of labeling. This labeling method can be used for other studies related to phenotype detection based on unstructured clinical notes.

## Appendix

Multimedia Appendix 1 Demographic features.

Multimedia Appendix 2 Examples for delirium sentences and always patterns.

Multimedia Appendix 3 Two query strategies.

Multimedia Appendix 4 Other delirium indicators.

Multimedia Appendix 5 Interrater agreement.

Multimedia Appendix 6 Data splitting.

## Abbreviations

AUC: area under the curve
BERT: Bidirectional Encoder Representations from Transformers
CAM: Confusion Assessment Method
EHR: electronic health record
GUI: graphical user interface
ICD: International Classification of Diseases
IRA: interrater agreement
LSTM: long short-term model
NIH: National Institutes of Health
NLP: natural language processing
RNN-LSTM: recurrent neural networks with LSTM units
ROC: receiver operating characteristic
SVM: Support Vector Machine
