# Supplementary material for: Identifying Patients With Delirium Based on Unstructured Clinical Notes: Observational Study
Source: JMIR Form Res. 2022 Jun 24;6(6):e33834. doi: 10.2196/33834 (PMC9270709; doi:10.2196/33834)
Supplement: Multimedia Appendix 1 [file formative_v6i6e33834_app1.docx]

**A1 demographic features**

**Table 1 Demographic features**

| dataset | Patients | Age  Mean  (sd) | Female  Number  (%) | Race | | | | |
| --- | --- | --- | --- | --- | --- | --- | --- | --- |
|  |  |  |  | White or  Caucasian  (%) | Black or African American  (%) | Hispanic or Latino  (%) | Asian  (%) | other  (%) |
| AED dataset | 852 | 59.80  (17.39) | 413  (48.47%) | 629  (73.83%) | 52  (6.10%) | 10  (1.17%) | 28  (3.28%) | 133  (15.61%) |
| GIFT dataset | 576 | 79.39  (8.67) | 411  (71.35%) | 503  (87.33%) | 10  (1.74%) | 3  (0.52%) | 15  (2.60%) | 45  (7.81%) |
| Dementia dataset | 802 | 78.63  (7.22) | 486  (60.59%) | 727  (90.65%) | 21  (2.62%) | 2  (0.25%) | 9  (1.12%) | 43  (5.36%) |
| Covid-19 dataset | 3429 | 63.62  (18.76) | 1567  (45.69%) | 2690  (78.45%) | 252  (7.35%) | 28  (0.82%) | 83  (2.42%) | 376  (10.97%) |
| NCC dataset | 1985 | 59.47  (18.89) | 902  (45.44%) | 926  (46.65%) | 343  (17.28%) | 0  (0.00%) | 84  (4.23%) | 632  (31.84%) |
| LTM dataset | 395 | 59.20  (17.63) | 165  (41.77%) | 301  (76.20%) | 28  (7.09%) | 1  (0.25%) | 11  (2.78%) | 54  (13.67%) |
| Control  dataset | 2477 | 43.67  (24.01) | 1375  (55.51%) | 1397  (56.40%) | 106  (4.28%) | 26  (1.05%) | 86  (3.47%) | 862  (34.80%) |
