# Supplementary material for: Identifying Patients With Delirium Based on Unstructured Clinical Notes: Observational Study
Source: JMIR Form Res. 2022 Jun 24;6(6):e33834. doi: 10.2196/33834 (PMC9270709; doi:10.2196/33834)
Supplement: Multimedia Appendix 2 [file formative_v6i6e33834_app2.docx]

**A2 examples for delirium sentences and always patterns**

Labels categorized sentences containing delirium keywords into three bins (examples given in Figure 1).

1. **Positive:** Sentences that indicate the likely presence of delirium (sentence 1,2).
2. **Negative:** Sentences that deny the presence of delirium. Often sentences with negative patterns contain negations, such as “no”, “negative”, “without” (sentence 3). However, often they do not (sentence 4). Therefore, detecting negative cases is not simply a negation detection task.
3. **Not relevant (Neither):** Sentences that contain delirium keywords, but neither confirm nor deny the presence of delirium (sentence 5,6).

**Fig. 1** examples for delirium sentences and always patterns
