# Supplementary material for: Identifying Patients With Delirium Based on Unstructured Clinical Notes: Observational Study
Source: JMIR Form Res. 2022 Jun 24;6(6):e33834. doi: 10.2196/33834 (PMC9270709; doi:10.2196/33834)
Supplement: Multimedia Appendix 3 [file formative_v6i6e33834_app3.docx]

**A3 two query strategies**

The two query strategies used in our implementation of Active Learning were as follows.

**Uncertainty based on entropy of prediction scores:** Denote the number of unlabeled sentences by $N$. The n-th unlabeled sentence is denoted $x_{n}$, where $1\leq n\leq N$. The label (Positive, Negative or Neither) of $x_{n}$ is denoted $y_{n,m}$, where $1\leq m\leq M$ is the index of the patterns. The classifier in the previous iteration is used to calculate the label probabilities for $x_{n}$ denoted $p_{n,m}=Pr(y_{n,m}|x_{n})$, where $\sum_{m=1}^{M} p_{n,m}=1, 1\leq n\leq N$. The entropy of the n-th unlabeled sentence $x_{n}$ is represented as

$$s_{n}=-\sum_{m=1}^{M} p_{n,m}\cdot\log p_{n,m} (1)$$

The normalized probability of sampling the n-th unlabeled sentence is calculated as

$$p_{n}^{\mathrm{sample}}=\frac{exp\left( s_{n} \right)}{\sum_{n=1}^{N} exp\left( s_{n} \right)} (2)$$

**Diversity based on embedding map:** In the embedding map, the similarity between unlabeled sentence $a_{n}$ and labeled sentence $b_{k}$ is represented as ^7^

$$\text{Similarity}\left( a_{n},b_{k} \right)=\frac{1}{{{||a}_{n} - b_{k}||}^{2}} (3)$$

Where $1\leq n\leq N$, $1\leq k\leq K$, and $K$ is the number of labeled sentences. The diversity for the unlabeled sentences $a_{n}$ is defined as

$$s_{n}=\text{Diversity}\left( a_{n} \right)=\frac{1}{\max_{1\leq k\leq K} \text{Similarity}\left( a_{n},b_{k} \right)}=\min_{1\leq k\leq K} {{||a}_{n} - b_{k}||}^{2} (4)$$

The normalized probability of sampling the n-th unlabeled sentence point $a_{n}$ is calculated via equation (2).
