# Supplementary material for: Identifying Patients With Delirium Based on Unstructured Clinical Notes: Observational Study
Source: JMIR Form Res. 2022 Jun 24;6(6):e33834. doi: 10.2196/33834 (PMC9270709; doi:10.2196/33834)
Supplement: Multimedia Appendix 4 [file formative_v6i6e33834_app4.docx]

**A4 other delirium indicators**

**ICD codes**: International Classification of Diseases codes are used by physicians for billing purposes. Following [^4^], we utilized the following ICD-9 codes for delirium: 293, 293.1, 292.81, 290.11, 290.3, 290.41, 291, 293.9, 780.09, 293.81, 293.82, 293.83, 293.84, 293.89, 290.12, 290.13, 290.43, 292.11, 292.12, 292.2, 780.02, 290.2, 290.42, 290.8, 290.9, 292, 292.82, 348.3, 348.31, 348.39, 349.82, 780.97. These ICD-9 codes were mapped to corresponding ICD-10 codes in our EHR.

**Medications**: We hypothesized that, to the extent that NLP delirium detections are valid, they should be associated with use of delirium-related medications. We identified over 30 medications commonly associated with delirium, either because they are used to treat symptoms of delirium or are reported to contribute to delirium. These included typical and atypical antipsychotics, benzodiazepines, cholinesterase inhibitors, and trazadone (Table 2).

**Restraints & Sitter Orders**: Delirium increases the likelihood of physical restraint use and the use of sitters, particularly for patients at risk of self-injury (e.g. getting out of bed and falling). We hypothesized that NLP delirium detection, if valid, should be associated with use of orders for restraints and sitters.

**CAM**: The Confusion Assessment Method (CAM) is a standardized evidence-based tool that enables non-psychiatrically trained clinicians to identify and recognize delirium quickly and accurately in both clinical and research settings.^11^ There is also a CAM-ICU version for use with non-verbal mechanically ventilated patients. We hypothesized that, if NLP delirium detection are valid, they should be associated with positive in-person CAM assessments. CAM assessments were available in the LTM dataset. These were performed at the bedside by study staff using the CAM-ICU, which assesses four features: 1) Acute change/fluctuating course, 2) Inattention, 3) Altered level of consciousness, 4) Disorganized thinking; with a positive result defined by the presence of features 1 and 2, with the additional presence of either feature 3 or 4. For patients comatose at evaluation, CAM-ICU was assigned a positive result. Each delirium evaluation was conducted by a single member of the study team. Staff were trained to perform assessments through a combination of didactics, literature review, in person case reviews, and ongoing discussions.

**Mortality**: Delirium is known to be associated with increased risk of in-hospital mortality.^12^ We hypothesized that, if NLP delirium detection are valid, they should be associated with increased risk of death in the hospital. As a further check of validity, within the GIFTS dataset, we also further assessed whether the risk of in-hospital death increases with the number of days with delirium as determined by NLP scanning of notes.

**Table 2, Medications for delirium**

| **Category** | **Generic Name** | **Brand Name** |
| --- | --- | --- |
| Antipsychotics | Benperidol | Anquil |
|  | Chlorpromazine | Largactil |
|  | Flupentixol | Depixol |
|  | Fluphenazine | Modecate |
|  | Haloperidol | Haldol |
|  | Levomepromazine | Nozinan |
|  | Perphenazine | Fentazin |
|  | Pimozide | Orap |
|  | Promazine | Sparine |
|  | Sulpiride | Dolmatil, Sulpor |
|  | Trifluoperazine | Stelazine |
|  | Zuclopenthixol | Clopixol |
| Atypical Antipsychotics | Amisulpride | Solian |
|  | Aripiprazole | Abilify, Abilify Maintena |
|  | Clozapine | Clozaril, Denzapine, Zaponex |
|  | Risperidone | Risperdal & Risperdal Consta |
|  | Olanzapine | Zyprexa |
|  | Quetiapine | Seroquel |
|  | Paliperidone | Invega, Xeplion |
| Benzodiazepines | Alprazolam | Xanax |
|  | Chlordiazepoxide | Librium |
|  | Clonazepam | Klonopin |
|  | Clorazepate | Tranxene |
|  | Diazepam | Valium |
|  | Estazolam | Prosom |
|  | Flurazepam | Dalmane |
|  | Lorazepam | Ativan |
|  | Midazolam | Versed |
|  | Oxazepam | Serax |
|  | Temazepam | Restoril |
|  | Triazolam | Halcion |
|  | Quazepam | Doral |
| Cholinesterase Inhibitors | Donepezil | Aricept |
|  | Galantamine | Razadyne |
|  | Rivastigmine | Exelon |
| Trazodone | Trazodone | Desyrel |
