# Supplementary material for: Identifying Patients With Delirium Based on Unstructured Clinical Notes: Observational Study
Source: JMIR Form Res. 2022 Jun 24;6(6):e33834. doi: 10.2196/33834 (PMC9270709; doi:10.2196/33834)
Supplement: Multimedia Appendix 5 [file formative_v6i6e33834_app5.docx]

**A5 Inter-Rater Agreement**

The pairwise Inter-Rater Agreement is used to measure agreement between human and human (model) for each category. The pairwise agreement between the i-th expert (human) and the j-th expert (human or model) for the m-th category is defined as

$$A_{i,j}(m)=\frac{1}{2}\left\{ \frac{\sum_{k=1}^{K} \left[ \mathbb{l}\left( v_{i,k},m \right)\mathbb{\cdot l}\left( v_{j,k},m \right) \right]}{\sum_{k=1}^{K} \mathbb{l}\left( v_{i,k},m \right)}+\frac{\sum_{k=1}^{K} \left[ \mathbb{l}\left( v_{i,k},m \right)\mathbb{\cdot l}\left( v_{j,k},m \right) \right]}{\sum_{k=1}^{K} \mathbb{l}\left( v_{j,k},m \right)} \right\}. (5)$$

where $v_{i,k}$ is the result that the i-th expert for the k-th sentence, and

$$\mathbb{l}\left( v_{i,k},m \right)=\left\{ \begin{matrix} 1 if v_{i,k}=m \\ 0 otherwise \end{matrix} \right. (6)$$

The average pairwise IRA between experts, called expert-expert agreement (EEA), is defined as

$EEA=\frac{1}{I\left( I-1 \right)}\sum_{i=1}^{I} \sum_{j=1,j\neq i}^{I} A_{i,j}$, (7)

The average agreement between the algorithm and experts, called expert-algorithm agreement (EAA), is defined as

$EAA=\frac{1}{I}\sum_{i=1}^{I} A_{i,\mathrm{model}}$ (8)
