# Supplementary material for: Identifying Patients With Delirium Based on Unstructured Clinical Notes: Observational Study
Source: JMIR Form Res. 2022 Jun 24;6(6):e33834. doi: 10.2196/33834 (PMC9270709; doi:10.2196/33834)
Supplement: Multimedia Appendix 6 [file formative_v6i6e33834_app6.docx]

**A6 data splitting**

AED

GIFTS

Dementia

Control

Covid-19

NCC

LTM

60% training, 20% validation, 20% test

70% Training, 30% validation

**datasets for labeling sentences and developing always patterns**

test

70% Training, 30% validation

Training

Validation

test

**Internal Test**

**External Test /**

**Association**

**Evaluation for LTM**

**Association**

**Evaluation for NCC**

**Fig. 2** data splitting
